# Prevention of hepatorenal toxicity with *Sonchus asper *in gentamicin treated rats

**DOI:** 10.1186/1472-6882-11-113

**Published:** 2011-11-15

**Authors:** Muhammad R Khan, Iram Badar, Aisha Siddiquah

**Affiliations:** 1 Department of Biochemistry, Faculty of Biological Sciences, Quaid-i-Azam University Islamabad, 44000, Pakistan

## Abstract

**Background:**

*Sonchus asper *possesses antioxidant capacity and is used in liver and kidney disorders. We have investigated the preventive effect of methanolic extract of *Sonchus asper *(SAME) on the gentamicin induced alterations in biochemical and morphological parameters in liver and kidneys of Sprague-Dawley male rat.

**Methods:**

Acute oral toxicity studies were performed for selecting the therapeutic dose of SAME. 30 Sprague-Dawley male rats were equally divided into five groups with 06 animals in each. Group I received saline (0.5 ml/kg bw; 0.9% NaCl) while Group II administered with gentamicin 0.5 ml (100 mg/kg bw; i.p.) for ten days. Animals of Group III and Group IV received gentamicin and SAME 0.5 ml at a dose of 100 mg/kg bw and 200 mg/kg bw, respectively while Group V received only SAME at a dose of 200 mg/kg bw. Biochemical parameters including aspartate transaminase (AST), alanine transaminase (ALT), alkaline phosphatase (ALP), lactate dehydrogenase (LDH), γ-glutamyltransferase (γ-GT), total cholesterol, triglycerides, total protein, albumin, creatinine, blood urea nitrogen (BUN), total bilirubin and direct bilirubin were determined in serum collected from various groups. Urinary out puts were measured in each group and also assessed for the level of protein and glucose. Lipid peroxides (TBARS), glutathione (GSH), DNA injuries and activities of antioxidant enzymes; catalase (CAT), peroxidase (POD) and superoxide dismutase (SOD) were determined in liver and renal samples. Histopathological studies of liver and kidneys were also carried out.

**Results:**

On the basis of acute oral toxicity studies, 2000 mg/kg bw did not induce any toxicity in rats, 1/10^th ^of the dose was selected for preventive treatment. Gentamicin increased the level of serum biomarkers; AST, ALT, ALP, LDH, γ-GT, total cholesterol, triglycerides, total protein, albumin, creatinine, BUN, total and direct bilirubin; as were the urinary level of protein, glucose, and urinary output. Lipid peroxidation (TBARS) and DNA injuries increased while GSH contents and activities of antioxidant enzymes; CAT, POD, SOD decreased with gentamicin in liver and kidney samples. SAME administration, dose dependently, prevented the alteration in biochemical parameters and were supported by low level of tubular and glomerular injuries induced with gentamicin.

**Conclusion:**

These results suggested the preventive role of SAME for gentamicin induced toxicity that could be attributed by phytochemicals having antioxidant and free radical scavenging properties.

## Background

Gentamicin is an aminoglycoside, natural or semisynthetic antibiotic clinically used against Gram negative bacteria because of its efficacy and failure to increase bacterial resistance. However, the main problem associated with gentamicin is the cause of nephrotoxicity at its therapeutic doses and appeared to be a major cause of nonoliguric acute renal failure [1]. Gentamicin is excreted through kidneys without degradation or metabolic changes, with 5 to 10% dose is concentrated in proximal tubules vastly exceeding the concurrent serum concentration [2]. Although its exact role in kidney dysfunction is not apparent, it is suggested that the selective accumulation of gentamicin in kidney cortex can induce oxidative stress and cause lipid peroxidation [3]. Additionally, it can mediate the generation of reactive oxygen species (ROS) such as hydroxyl radical, hydrogen peroxide and superoxide anion mostly in mitochondria that can induce renal injuries [3].

Gentamicin causes the activation of platelet activation factor ensuing local vasoconstriction and thus reduces the renal blood flow and glomerular filtration rate [4]. Gentamicin induced renal injuries are mostly localized to the proximal tubules because of association of gentamicin with polyanionic inositol phospholipids and *megalin*, a receptor, for uptake of gentamicin [5,6]. Gentamicin resists the degradation of phospholipids that compromised the lysosomal membrane integrity and eventually the leakage of enzymes. Gentamicin behaves as iron chelator and iron-gentamicin complexes are thought to be involved in free radical formation [7]. Iron, released from the renal cortex mitochondria on account of excessive oxidative stress induced with gentamicin, act as a putative enhancer of gentamicin induced oxidative stress. Oxidative stress is mainly regulated by the cellular enzymatic (catalase, superoxide dismutase, glutathione peroxidase) and nonenzymatic (glutathione, ascorbic acid, α-tocopherol) factors [7].

Recent studies have shown that natural antioxidants obtained from different alternative systems of medicine display a wide range of biological activities. Various alternatives possessing antioxidant properties have been used in order to minimize gentamicin induced oxidative stress in animal models. Many plant extracts have been reported to be effective in ameliorating organ toxicities [8-11]. Prevention of nephrotoxicity induced with gentamicin in rat by grape seed extract might be attributed by the presence of polyphenolic compounds [8]. Green tea extract possesses catechin and other polyphenolic compounds; prevents the gentamicin induced nephrotoxicity in rat [10,11]. *Momordica dioica *extract also prevented the toxic insult caused by gentamicin in rat [12]. Coenzyme Q10 played a significant role in protecting cells from oxidative stress induced with gentamicin and other agents [9,13]. Another antioxidant that possibly deactivates ROS is curcumin. This chemical is obtained from *Curcuma longa*; decreased the oxidative stress induced with gentamicin and is also found beneficial against AIDS [14,15]. Erdosteine, a mucolytic agent also impart free radical scavenging and antioxidant activities against gentamicin induced nephrotoxicity and other pharmacological or noxious agents [16,17]. Experimental data suggest that renoprotective effects of plant extracts or chemicals are mainly attributed through antioxidant/scavenging of free radicals, metal chelation and stabilizing the membranous system [11].

*Sonchus asper *grows wild in fields and also in wastelands in Pakistan. This plant is locally utilized in renal disorders [18]. Marked renoprotective effects of methanol extract of *Sonchus asper *against carbon tetrachloride induced injuries are reported in our previous studies [19]. Similarly methanolic extract of *Sonchus asper *is found beneficial in decreasing the oxidative stress induced with potassium bromated in cardiovascular system of rat [20]. Chemical characterization of *Sonchus asper *revealed the presence of polyphenolics such as rutin, quercetin, catechin and myricetin [21,22]. Since liver and kidneys are involved in the metabolism and excretion of xenobiotics, these may be more prone to various pathological conditions. The inability of conventional medicine to minimize the gentamicin induced oxidative damage has contributed for the use of complementary medicine. However, no studies have been performed on the antioxidant potential of *Sonchus asper *against the oxidative stress caused by gentamicin. Therefore, this study was designed to investigate whether the methanolic extract of *Sonchus asper *ameliorates the hepatorenal toxicity induced with gentamicin in rat model.

## Methods

### Preparation of plant extract

Whole plant (leaves, stem, flowers, seeds and roots) of *Sonchus asper *at maturity was collected from District Rawalpindi and after identification a specimen was submitted at herbarium of Pakistan, Quaid-i-Azam University Islamabad Pakistan. Shade dried powdered (2 kg) was extracted twice with methanol for three days. The methanolic solution was filtered and dried in rotary evaporator to get the crude methanol extract of *Sonchus asper *(SAME).

### Acute toxicity studies

Acute oral toxicity studies were carried out on 150-200 g body weight of Sprague-Dawley male rats according to defined doses (5, 50, 300, 2000 mg/kg bw) to the overnight fasted rats but water was provided *ad libitum*. The onset and sign of toxicity were noted for each dose for three days. Since the dose of 2000 mg/kg bw did not induce any sign of toxicity, it was selected for the preventive effects against the gentamicin treatment to rats [23].

### Animals and experimental protocols

30 Sprague-Dawley male rats (160-200 g) were acquired from National Institute of Health (NIH) Islamabad. Animals were kept at the Primate Facility maintained at Quaid-i-Azam University Islamabad in ordinary cages for two weeks at room temperature of 25 ± 3°C with a 12 h dark/light cycle for acclimatization. Animals had free access to food and water *ad libitum*. The experimental protocol and procedures used in this study were approved by the Ethics committee of the Quaid-i-Azam University, Islamabad, Pakistan for the care and use of laboratory animals. Dose of gentamicin was given as described previously [24]. Rats were divided into five groups (n = 06 rats per group) with the following protocols:

Group I: animals received saline (0.5 ml/kg bw; 0.9% NaCl) orally

Group II: gentamicin (0.5 ml) administered at a dose of 200 mg/kg bw intraperitoneally

Group III: rats received gentamicin and 0.5 ml of SAME (100 mg/kg bw) orally through a feeding tube by lowering in the stomach.

Group IV: animals treated with gentamicin and 0.5 ml SAME (200 mg/kg bw; orally)

Group V: animals received 0.5 ml of SAME only (200 mg/kg bw; orally)

All these treatments were given to rats for ten days. Animals were anesthetized with ether, 24 h after the last treatment, blood was collected through cardiac puncture, centrifuged at 1500 × g for 10 min, at 4°C to collect serum.

### Histopathological studies of liver and kidneys

Liver and kidneys were perfuse with cold saline at 4°C and excised immediately. For histological studies a portion of liver and kidney was fixed in sera (absolute alcohol 60%, formaldehyde 30%, glacial acetic acid 10%).

### Liver function tests

Analysis of serum was carried out for alanine transaminase (ALT), aspartate transaminase (AST), alkaline phosphatase (ALP), lactate dehydrogenase (LDH) and gamma glutamyltransferase (γ-GT) with standard AMP diagnostic kits (Stattogger Strasse 31b 8045 Graz, Austria).

### Serum analysis for biochemical studies

Level of blood urea nitrogen (BUN), creatinine, total protein, albumin, total bilirubin, direct bilirubin, total cholesterol, triglycerides in serum was determined by using standard AMP diagnostic kits (Stattogger Strasse 31b 8045 Graz, Austria).

### Antioxidant enzyme assays in hepatic and renal samples

Hepatic and renal tissues were homogenized in 10 volume of phosphate buffer (100 mM) having EDTA (pH 7.4; 1 mM), centrifuged (12000 × g) at 4°C for 30 min. Supernatant was used to estimate the protein contents by using bovine serum albumin (BSA) as standard [25]. Catalase (CAT) and peroxidase (POD; 1.11.1.7) activity was estimated by following the method of Chance and Maehly (1955) [26] in liver and renal homogenates. In this experiment hydrogen peroxide was used as substrate for catalase activity while guaiacol was used for peroxidase activity. However, superoxide dismutase (SOD) activity was determined according to the procedure described previously [27].

### Estimation of renal glutathione (GSH) and lipid peroxidation (TBARS)

Hepatic and renal glutathione (GSH) contents were determined according to the method described previously [28] by using DTNB (5,5-dithiobis-2-nitrobenzoic acid) as substrate. Level of lipid peroxidation (TBARS) in renal homogenates was determined according to the method described by Iqbal et al. (1996) [29] using thiobarbituric acid as reacting substrate.

### DNA fragmentation assay

For DNA fragmentation estimation, liver and renal tissue was homogenized in 10 volumes of a TE solution pH 8.0 (5 mM Tris-HCl, 20 mM EDTA), 0.2% triton X-100 and centrifuged at 27,000 × *g *for 20 min to separate the intact chromatin (pellet A) from the fragmented (supernatant B) [30]. 1.0 ml aliquot of each sample was centrifuged at 27,000 × g for 20 min. Quantification of DNA for the intact chromatin and fragmented chromatin was determined by using freshly prepared DPA (Diphenylamine) solution and optical density was recorded at 620 nm with SmartSpec Spectrophotometer. Quantity of fragmented DNA was estimated by using the following formula:

$$\%\text{ fragmented DNA}=\text{B}\times \text{1}00∕\left(\text{A + B}\right)$$

### DNA ladder assay

DNA was isolated from the liver and renal tissues by following the method described previously [30]. 5 μg of DNA of both organs was loaded in 1.5% agarose gel containing 1.0 μg/ml ethidium bromide. After electrophoresis gel was studied under gel doc system and was photographed.

### Analysis of urine

After the last treatment animals were shifted to metabolic cages for 24 h in order to collect their urine individually and to estimate urine volume. Urine samples were assayed for glucose and protein by using standard diagnostic kits (MediScreen Orgenics, France).

### Statistical analysis

Data recorded was analyzed for the mean and standard deviation in each group. Differences between groups were determined by performing one way analysis of variance and *post hoc *comparison in terms of least significance difference (LSD) at 0.05 and 0.01 by using the Statistical Package for Social Sciences (SPSS) software for Windows (version 14.0).

## Results

### Effect of SAME on liver marker enzymes

Gentamicin increased the level of biochemical parameters; AST, ALT, ALP, LDH and γ-GT in serum as compared to that of the control group (Table 1). Treatment of SAME in combination with gentamicin reduced the serum level of AST, ALT, ALP, LDH and γ-GT, dose dependently, to that of the gentamicin treated group. Treatment of SAME (200 mg/kg bw) alone did not induce significant (P>0.05) change in the level of liver marker enzymes as compared to control group.

**Table 1:** Effects of SAME on liver marker profile in rat

Treatment	AST U/l	ALT U/l	ALP U/l	LDH U/l	γ-GT U/l
Control (Saline, 0.9% NaCl)	83.3 ± 9.3A	34.3 ± 4.0A	246.7 ± 16.3A	50.8 ± 6.9A	69.8 ± 6.8A
GTM (100 mg/kg bw)	182.6 ± 18.3D	68.9 ± 8.0D	366.3 ± 26.7E	64.3 ± 7.0C	94.2 ± 8.8D
GTM + SAME (100 mg/kg bw)	148.9 ± 17.6C	63.8 ± 5.6C	311.3 ± 17.8D	59.5 ± 6.2B	84.6 ± 7.6C
GTM + SAME (200 mg/kg bw)	122.1 ± 10.6B	51.0 ± 6.9B	263.7 ± 19.0C	49.0 ± 4.8A	79.5 ± 6.8BC
SAME (200 mg/kg bw)	83.5 ± 6.8A	35.6 ± 5.9A	240.7 ± 13.8A	49.7 ± 6.3A	72.6 ± 7.3A

Mean ± SD (n = 06). A-D, Means not sharing common letters within a column are significantly (P < 0.01) different from each other

GTM, gentamicin; SAME, methanol extract of *Sonchus asper*

### Effect of SAME on serum biochemistry

Injection of gentamicin to rats for ten days significantly (P < 0.01) decreased the serum level of total protein and albumin while the level of total cholesterol, triglycerides, total and direct bilirubin increased than that of the control group (Table 2). In addition, altered level of these parameters induced with gentamicin in serum was restored, dose dependently, with the simultaneous treatment of SAME to rats than that of the control group. However, administration of SAME alone to rats did not cause any significant (P>0.05) change in the parameter studied as compared to the control group (Table 2).

**Table 2:** Effects of SAME on serum chemistry in rat

Treatment	Total protein (mg/dl)	Albumin (mg/dl)	Triglycerides (mg/dl)	Total cholesterol (mg/dl)	Total bilirubin (mg/dl)	Direct bilirubin (mg/dl)	Urea (mg/dl)	Creatinine (mg/dl)
Control (Saline, 0.9% NaCl)	42.5 ± 4.7A	22.7 ± 3.5A	12.8 ± 2.8A	6.8 ± 0.84A	1.51 ± 0.36A	0.87 ± 0.14A	33.5 ± 5.7A	0.78 ± 0.16A
GTM (100 mg/kg bw)	33.7 ± 3.7D	16.4 ± 2.3D	18.6 ± 2.0B	12.0 ± 2.95D	2.83 ± 0.45D	1.17 ± 0.44E	56.2 ± 6.6C	3.7 ± 0.60C
GTM + SAME (100 mg/kg bw)	35.7 ± 4.1C	18.4 ± 2.8C	17.2 ± 2.2B	10.1 ± 1.73CD	1.69 ± 0.34C	1.03 ± 0.16C	43.2 ± 5.2BC	2.6 ± 0.65B
GTM + SAME (200 mg/kg bw)	40.2 ± 5.6B	21.1 ± 3.2AB	13.1 ± 2.2A	8.8 ± 1.50B	1.67 ± 0.24C	0.97 ± 0.17B	40.6 ± 6.2B	1.2 ± 0.26A
SAME (200 mg/kg bw)	43.2 ± 4.2A	23.2 ± 2.9A	12.1 ± 1.4A	7.2 ± 1.69A	1.49 ± 0.36A	0.83 ± 0.16A	32.4 ± 4.6A	0.84 ± 0.32A

Mean ± SD (n = 06). A-D, Means not sharing common letters within a column are significantly (P < 0.01) different from each other.

GTM, gentamicin; SAME, methanol extract of *Sonchus asper*

### Effects of SAME on urinary protein, glucose and urine output in rat

Effect of gentamicin and gentamicin plus SAME on urinary protein, glucose and urine output are shown in Table 3. Urinary excretion of protein, glucose and urinary output significantly increased with gentamicin than that of the control group. Administration of SAME in combination with gentamicin ameliorated the toxicity of gentamicin and the level of protein, glucose and urinary output significantly reduced, as compared to that of the gentamicin treated group. However, non significant (P>0.05) alteration in the level of urinary protein, glucose and in urine output was recorded with the SAME administration alone as compared to the control group.

**Table 3:** Effects of SAME on urinary profile of rat

Treatment	Urinary volume (ml/day)	Urinary glucose (mg/L/day)	Urinary protein (mg/L/day)
Control (Saline, 0.9% NaCl)	7.4 ± 1.24A	9.4 ± 1.2A	16.5 ± 2.62A
GTM (100 mg/kg bw)	14.6 ± 3.24C	53.8 ± 7.4D	38.8 ± 7.16B
GTM + SAME (100 mg/kg bw)	11.5 ± 2.76B	42.9 ± 5.4C	28.9 ± 4.32B
GTM + SAME (200 mg/kg bw)	9.3 ± 2.22A	25.5 ± 3.4B	19.8 ± 3.16A
SAME (200 mg/kg bw)	8.3 ± 1.63A	8.4 ± 1.3A	16.31 ± 4.25A

Mean ± SD (n = 06). A-D, Means not sharing common letters within a column are significantly (P < 0.01) different from each other.

GTM, gentamicin; SAME, methanol extract of *Sonchus asper*

### Effects of SAME on hepatorenal antioxidant enzymes of rat

Effects of gentamicin, and gentamicin plus SAME on hepatorenal antioxidant defense system such as CAT, POD and SOD are shown in Table 4 and 5. Administration of gentamicin significantly (P < 0.01) decreased the activities of both hepatic and renal CAT, POD and SOD as compared to control group. Treatment of SAME along with gentamicin increased the activity level of CAT, POD and SOD, dose dependently, as compared to the gentamicin treated group. However, non significant (P>0.05) change was recorded with the treatment of SAME alone as compared to control group.

**Table 4:** Effects of SAME on liver antioxidant enzymes of rat

Treatment	CAT (U/mg protein)	POD (U/mg protein)	SOD (U/mg protein)
Control (Saline, 0.9% NaCl)	10.3 ± 1.5A	12.7 ± 1.2A	21.2 ± 2.5A
GTM (100 mg/kg bw)	5.8 ± 1.0B	6.8 ± 0.9D	13.2 ± 1.6C
GTM + SAME (100 mg/kg bw)	8.8 ± 1.0A	8.4 ± 1.2C	16.7 ± 1.2B
GTM + SAME (200 mg/kg bw)	9.8 ± 1.2A	10.4 ± 1.5B	20.2 ± 1.9A
SAME (200 mg/kg bw)	10.3 ± 0.9A	12.8 ± 1.3A	22.3 ± 1.6A

Mean ± SD (n = 06). A-D, Means not sharing common letters within a column are significantly (P < 0.01) different from each other.

GTM, gentamicin; SAME, methanol extract of *Sonchus asper*

**Table 5:** Effects of SAME on renal antioxidant enzymes of rat

Treatment	CAT (U/mg protein)	POD (U/mg protein)	SOD (U/mg protein)
Control (Saline, 0.9% NaCl)	4.36 ± 0.8A	7.42 ± 1.2A	16.25 ± 3.93A
GTM (100 mg/kg bw)	1.46 ± 0.46D	3.84 ± 0.6D	8.80 ± 1.10B
GTM + SAME (100 mg/kg bw)	2.35 ± 0.7C	4.59 ± 1.4C	10.90 ± 2.22B
GTM + SAME (200 mg/kg bw)	3.30 ± 0.4B	5.53 ± 1.4B	14.68 ± 3.60A
SAME (200 mg/kg bw)	4.31 ± 0.7A	7.14 ± .9A	16.31 ± 2.85A

Mean ± SD (n = 06). A-D, Means not sharing common letters within a column are significantly (P < 0.01) different from each other.

GTM, gentamicin; SAME, methanol extract of *Sonchus asper*

### Effects of SAME on hepatorenal TBARS, GSH and DNA fragmentation in rat

Table 6 and 7 show the effects of SAME on TBARS, GSH and DNA fragmentation in liver and renal samples. Administration of gentamicin significantly (P < 0.01) decreased the level of GSH while increased (P < 0.01) the level of TBARS and DNA fragmentation as compared to the control group. Administration of SAME in combination with gentamicin significantly reversed the level of GSH, TBARS and DNA injuries as compared to gentamicin treated group in a dose dependent manner in both the liver and kidney samples. Treatment of SAME alone did not cause any significant (P>0.05) change in the level of GSH, TBARS and DNA injuries in renal samples.

**Table 6:** Effects of SAME on hepatic TBARS, GSH and DNA fragmentation of rat

Treatment	TBARS (nM /mg protein)	GSH (μM/g tissue)	DNA fragmentation%
Control (Saline, 0.9% NaCl)	76.9 ± 3.0A	0.74 ± 0.02A	0.48 ± 0.08A
GTM (100 mg/kg bw)	137.4 ± 6.0D	0.48 ± 0.03D	1.34 ± 0.13D
GTM + SAME (100 mg/kg bw)	116.6 ± 5.4C	0.59 ± 0.03C	1.11 ± 0.10C
GTM + SAME (200 mg/kg bw)	104.9 ± 4.4B	0.68 ± 0.04B	0.88 ± 0.09B
SAME (200 mg/kg bw)	76.1 ± 4.1A	0.76 ± 0.03A	0.40 ± 0.08A

Mean ± SD (n = 06). A-D, Means not sharing common letters within a column are significantly (P < 0.01) different from each other.

GTM, gentamicin; SAME, methanol extract of *Sonchus asper*

**Table 7:** Effects of SAME on renal TBARS, GSH and DNA fragmentation of rat

Treatment	TBARS (nM /mg protein)	GSH (μM/g tissue)	DNA fragmentation%
Control (Saline, 0.9% NaCl)	58.77 ± 6.4AB	0.82 ± 0.02A	0.21 ± 0.09A
GTM (100 mg/kg bw)	81.07 ± 7.92D	0.56 ± 0.03D	2.14 ± 0.11C
GTM + SAME (100 mg/kg bw)	70.48 ± 6.78C	0.67 ± 0.03C	1.62 ± 0.28C
GTM + SAME (200 mg/kg bw)	61.10 ± 5.82B	0.76 ± 0.04B	0.95 ± 0.14B
SAME (200 mg/kg bw)	59.62 ± 7.03AB	0.84 ± 0.03A	0.22 ± 0.04A

Mean ± SD (n = 06). A-D, Means not sharing common letters within a column are significantly (P < 0.01) different from each other.

GTM, gentamicin; SAME, methanol extract of *Sonchus asper*

### Effects of SAME on DNA ladder assay

**Figure 1:**
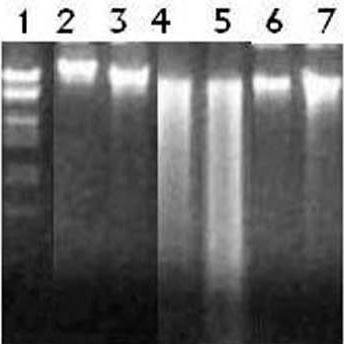
**Agarose gel showing DNA damages by gentamicin and preventive effects of SAME in different groups**. Lane from left (1) DNA marker (2) kidney control (3) liver control (4) gentamicin 100 mg/kg bw of kidney sample (5) gentamicin 100 mg/kg bw of liver sample (6) gentamicin 100 mg/kg bw + SAME 200 mg/kg bw of kidney sample (6) gentamicin 100 mg/kg bw + SAME 200 mg/kg bw of liver sample

DNA ladder assay showed a continuous banding pattern of fragmented DNA induced with gentamicin in both the organs; liver and kidneys (Figure 1). DNA injuries were comprehensively eliminated by the SAME treatment in liver and kidney samples. However, DNA fragmentation was not recorded in the control samples of liver and kidneys.

### Histopathological studies of liver

**Figure 2:**
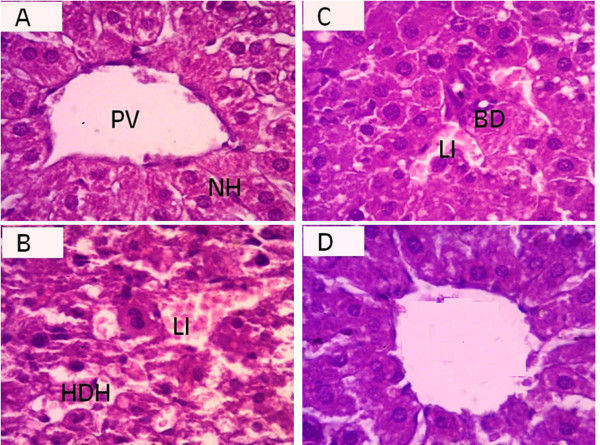
**Histopathology showing the alterations induced with gentamicin and preventive effects of SAME in hepatic tissues of rat**. (A) Hepatic section showing the normal architecture of portal vein (PV) and normal hepatocytes (NH) arranged in trabaculae. (B) Gentamicin (100 mg/kg bw) induced injuries in liver with severe level of leucocytes infiltration (LI) and hydropic degeneration of hepatocytes (HDH). (C) SAME (100 mg/kg bw) prevents injuries of gentamicin; medium level of leucocytes infiltration (LI) and bile ductile (BD). (D) SAME (200 mg/kg bw) prevents injuries of gentamicin; with normal hepatocytes and are arranged in trabaculae.

Liver of animal in the control group showed normal hepatic architecture, where the hepatocytes are arranged around the central vein and alternate with blood sinusoids. Each hepatic cell possesses a limiting membrane, centrally placed large nucleus and prominent nucleoli (Figure 2 A). Microscopic examination of animal livers treated with gentamicin depicted marked hepatocellular changes, dilated and congested sinusoids with hemorrhage (Figure 2B). Treatment of SAME along with gentamicin exhibited reversal of these changes (Figure 2C, D). However, preventive effects were more pronounced at higher dose of SAME (200 mg/kg bw). Light microscopic examination of H&E stained slides of the group having 200 mg/kg bw of SAME alone showed normal architecture of liver hepatic cords and plates radiating out from the central vein (Figure 2E).

### Histopathological studies of kidneys

**Figure 3:**
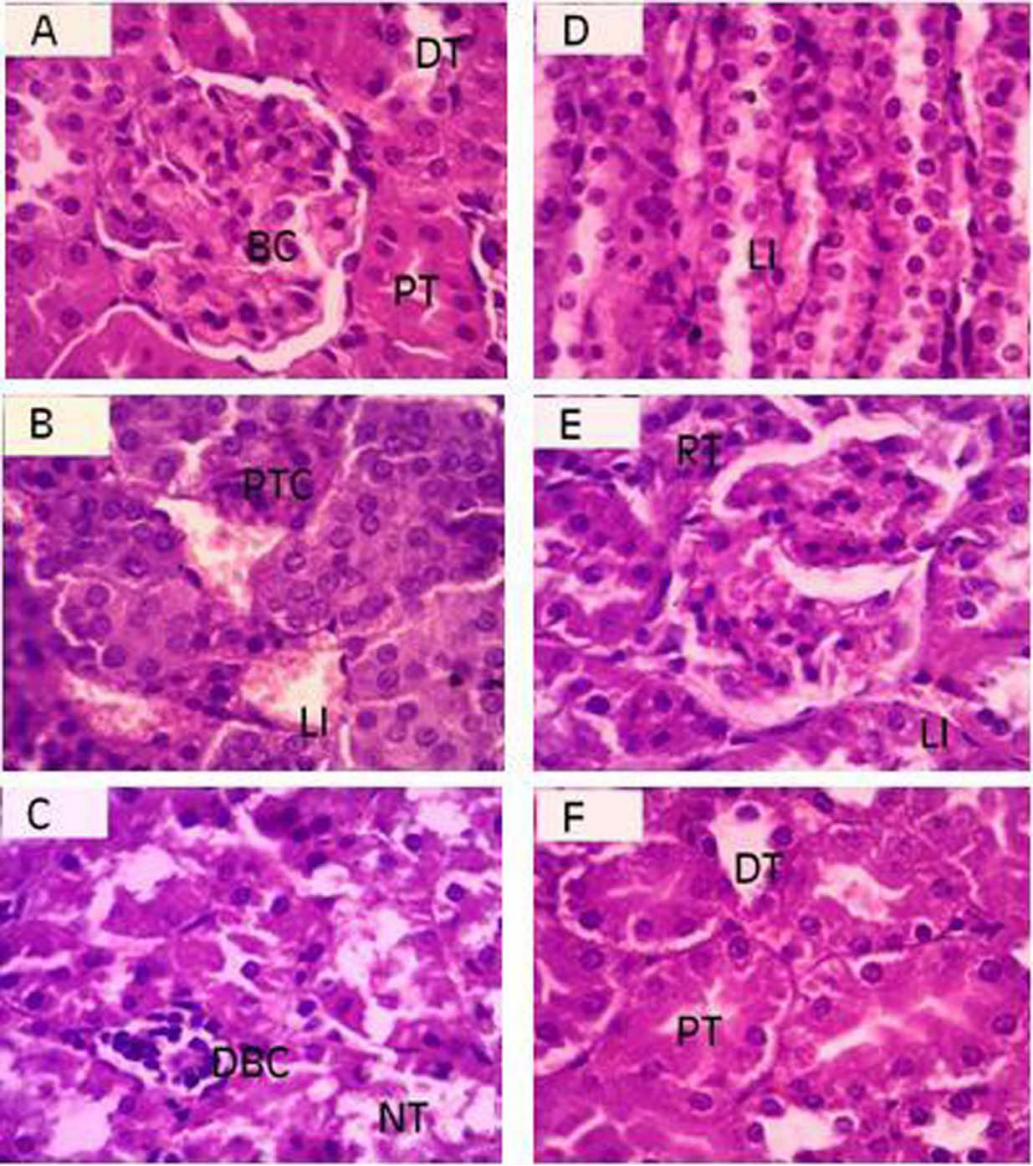
**Microphotographs of different experimental groups showing the histopathology of renal tissues**. (A) Normal structure of glomerulus, Bowman capsule (BC), proximal tubules (PT) and distal tubules (DT). (B) Alterations induced with gentamicin (100 mg/kg bw) in renal tissues; congested proximal tubules (PTC), leucocytes infiltration (LI). (C) Degeneration of bowman capsule and glomerulus (DBC), tubular necrosis (NT) with gentamicin nephrotoxicity. (D) Gentamicin induced leucocyte infiltration in medullary region (LI). (E) Regeneration of tubules (RT) and mild leucocyte infiltration (LI) with SAME (100 mg/kg bw) in combination with gentamicin. (F) normal architecture of proximal tubule (PT) and distal tubule (DT) with SAME (200 mg/kg bw) along with gentamicin.

Histopathological studies of kidneys showed the normal architecture including glomerulus, bowman capsule, distal and proximal convoluted tubules. Treatment of gentamicin markedly disrupted the histology as evidenced by the tubular degeneration, tubular congestion, tubular dilatation and glomerular injuries (Figure 3B,C). Prevention of nephropathological injuries was determined with SAME to gentamicin treated rats (Figure 3D).

## Discussion

Gentamicin is a therapeutic agent used against the life threatening infections in human, but it causes acute renal failure in 10-15% of the patients [31] while more than 30% of the patients showed the signs of nephrotoxicity which have received the gentamicin for more than 7 days [32,33]. In this study we have tested the hypothesis that SAME administration could prevent gentamicin induced hepatorenal toxicity in rat.

Treatment of gentamicin causes significant increase in the serum level of liver and kidney function markers such as creatinine, BUN, total cholesterol, triglycerides, total bilirubin, and direct bilirubin as compared to respective control indicating hepatorenal dysfunction. These injuries could be due to the production of free radicals and involvement of oxidative stress to hepatorenal toxicity caused by gentamicin treatment. Gentamicin may influence the various metabolic pathways of liver thereby enhancing the level of total cholesterol, triglycerides, total and direct bilirubin, BUN and creatinine in serum. Oxidative damage to kidneys by gentamicin may also contribute for the observed higher concentration of BUN and creatinine in serum. The same results have been reported in earlier studies where they have suggested the kidney dysfunction caused by gentamicin oxidative stress [8,13]. However, gentamicin treatment causes depletion in total protein and albumin, useful markers of liver function, might be depressed as a result of defective protein synthesis. Co-administration of SAME along with gentamicin caused significant decrease in the concentration of creatinine, BUN, total cholesterol, triglycerides, total bilirubin, and direct bilirubin while a significant increase in albumin and total protein of serum suggested the protective effects of SAME. Similar protective studies of different extracts against gentamicin have been reported previously [8,11,12].

Urinalysis also provides important clues about the functional status of kidneys. In this regard significantly higher quantity of urine volume, urinary glucose, and protein was recorded with gentamicin administration to rat for 10 days. At large scale excretion of gentamicin from kidneys, intracellular accumulation of gentamicin is confined primarily in the S_1 _and S_2 _segment of proximal convoluted tubules and can cause injuries [5,31,34]. These results are associated with significant excretion of urinary protein, glucosuria and urine output simulating the leakage and defects in reabsorption, and could be due to proximal tubular structural damage observed in this study. Histopathological examination of the gentamicin treated kidneys for 10 days indicated the presence of lesions in cortex with specifically in proximal convoluted tubules. Gentamicin probably binds to the negatively charged cationic phosphoinositides components of brush border of proximal tubules and associated with *megalin *that are taken up by lysosomes, results in disruption of various processes [6]. Use of SAME in combination with gentamicin for 10 days ameliorated the hepatorenal toxicity induced with gentamicin and resulted in the restoration of histopathology. Results obtained suggested that prevention of gentamicin induced renal toxicity with SAME at two different doses, might be due to the presence of various bioactive phytochemicals in the extract that may regulate and repair injured tissues. Similar results for these parameters were reported in other studies [8,9,12].

Gentamicin treatment causes hepatotoxicity as clearly indicated by the significant increase in serum level of ALT, AST, ALP, LDH and γ-GT, than those of control rats. Serum level of transaminases and ALP is generally considered as sensitive markers of liver function and their concentrations are increased in the serum because of their cytoplasmic nature and are thus released in blood by changing in the permeability of hepatocyte membranes. Increased level of LDH in serum in the present investigation apparently indicated the toxic effects of gentamicin in rat. The results obtained in this study are inconsistent with other reports [14]. Histopathological lesions observed in this study correlate the serum level of liver function enzymes concentration induced with gentamicin and the possible preventive effects of SAME treatment in gentamicin treated rats indicating the improvement in the functional status of liver [14].

Involvement of reactive oxygen species (ROS) in nephropathies of human are now characterized as a key element [1]. Gentamicin exposure to rats mediates the generation of ROS that play a significant role in the progression of hepatic and renal injuries including array of biomolecules such as membrane lipids, protein and nucleic acids especially in some organelles such as mitochondria and lysosomes of renal tissues [3]. Increased propagation of ROS mediates the peroxidation of polyunsaturated fatty acids, attached to biomembranes. Gentamicin induced lipid peroxidation impaired the cellular function and cause necrosis. In this study we observed a significant increase in the lipid peroxidation product (TBARS) with gentamicin. Upaganlawar et al. [9] have suggested that the development of oxidative stress may involve the release of iron from renal cortical mitochondria and gentamicin catalysis the reaction for the generation of ROS. Lipid peroxidation of renal tissues induced with gentamicin was prevented with SAME treatment. Amelioration of oxidative stress with green tea and grape seed extract was determined in previous studies [8,10,11]. Green tea constitutes epicatechin, epicatechingallate, epigallocatechin gallate and proanthrocyanidine potent antioxidants possibly decrease the oxidative stress induced with gentamicin [8,10,11]. Similarly the possible preventive effect of SAME, may be due to the presence of myricetin, quercetin, catechin and rutin [21,22] very active antioxidants and are known to play diverse biological roles [35,36]. The results obtained with SAME for protection of lipid peroxidation are consistent with previous studies [8,11,12].

In the present study alteration in the biochemical markers of oxidative stress was estimated as a response to gentamicin induced nephrotoxicity in rat. The nonenzymatic component of the self defense system; renal glutathione (GSH) and enzymatic components; CAT, SOD and POD was diminished in the rats treated with gentamicin as compared to the respective control group. Gentamicin enhances the generation of hydrogen peroxide, hydroxyl radical and superoxide anion in renal tissues [3] causing oxidative stress. Depletion in the CAT and SOD after gentamicin exposure has been reported in other studies suggested that oxidative stress is one of the cause of renal injuries induced with gentamicin toxicity in rat [8,11,12]. Treatment of SAME to rats in combination with gentamicin reversed all these alterations suggesting that SAME rendered its antioxidant properties by preventing or scavenging the ROS induced with gentamicin toxicity. Our results are in accord with other studies where antioxidant effects of other plants have been reported against gentamicin induced renal damage [8,11,12]. Similar protective effects of SAME were reported in our previous study where carbon tetrachloride was used as toxic agent [20]. Both carbon tetrachloride and gentamicin induced nephrotoxicity are considered to be due to the accumulation of free radicals. The protective mechanism of SAME could be similar in both kinds of xenobiotics.

The scavenging activity of polyphenolics was postulated to be governed by the position and number of the hydroxyl groups. Scavenging activity for peroxynitrite was enhanced with *ortho*-hydroxyl structures [37]. Flavonoids are oxidized by radicals, resulting in a more stable, less reactive radical. Flavonoids can also inhibit the activity of many enzymes such as xanthine oxidase, peroxidase and nitric oxide synthase, which are supposed to be involved in free radical generation, thereby resulting in decreased oxidative damage of macromolecules [38]. So the present results suggested that the probable mechanism of SAME for hepatorenal protection may be attributed for its free radical scavenging and antioxidant activity of its phenolics and flavonoids components [20,35]. Similar protective effects of *Sonchus asper *have been recorded in other studies [39].

Another effect of SAME is the protection of DNA of hepatorenal tissues which was significantly increased with gentamicin treatment to rats for 10 days. SAME probably reduces the oxidative damage to DNA by its putative antioxidant activities [16].

As the SAME is derived from herb, that is commonly used as vegetable and salad. Therapeutic effects of SAME may be attributed by the presence of different dietary polyphenolics. Due to the low solubility of flavonoid aglycones in water, to the short residence time of flavonoids in the intestine, and to the low coefficient of absorption, it is not possible for humans to suffer acute toxic effects from the consumption of flavonoids, with the exception of a rare occurrence of allergy [40]. We are of the opinion that the use of SAME in clinics may not cause any kind of side effects.

## Conclusion

Dietary polyphenols present in the SAME exhibit hepato and nephroprotective activities probably through free radical scavenging properties. Altered level of hepatic markers such as AST, ALT, ALP and LDH with gentamicin exposure was reversed towards normalization with SAME. Similarly, biochemical and urine parameters were also restored by SAME. These effects probably involve multiple molecular and biochemical mechanisms regulated by polyphenolics. Bioactive components of SAME ameliorated the oxidative damage and had increased the regenerative and reparative capacity of liver and kidneys. So it is mandatory to explore the bioactive chemicals in SAME in order to develop the therapeutics that have a promising role in the treatment of hepatorenal dysfunction diseases induced by xenobiotics.

## Competing interests

The authors declare that they have no competing interests.

## Authors' contributions

MRK has made substantial contribution to conception and design, interpretation of data, drafting and revising the manuscript for intellectual content. IB made significant contribution to acquisition of data, analysis, drafting of the manuscript. AS participated in the design and collection of data and analysis. All authors read and approved the final manuscript.

## Pre-publication history

The pre-publication history for this paper can be accessed here:

http://www.biomedcentral.com/1472-6882/11/113/prepub
